# Does long-term cadmium exposure influence the composition of pectic polysaccharides in the cell wall of *Medicago sativa* stems?

**DOI:** 10.1186/s12870-019-1859-y

**Published:** 2019-06-21

**Authors:** Annelie Gutsch, Kjell Sergeant, Els Keunen, Els Prinsen, Gea Guerriero, Jenny Renaut, Jean-Francois Hausman, Ann Cuypers

**Affiliations:** 1 Environmental Research and Innovation Department, Luxembourg Institute of Science and Technology, 5, avenue des Hauts-Fourneaux, 4362 Esch-sur-Alzette, Luxembourg; 2 Centre for Environmental Sciences, Hasselt University, Agoralaan building D, 3590 Diepenbeek, Belgium; 3 Integrated Molecular Plant Research, Department of Biology, University of Antwerp, Groenenborgerlaan 171, 2020 Antwerp, Belgium

**Keywords:** Long-term cadmium exposure, *Medicago sativa*, Label-free protein quantification, Gene expression, Cell wall, Lignin, Pectin methylesterase

## Abstract

**Background:**

The heavy metal cadmium (Cd) accumulates in the environment due to anthropogenic influences. It is unessential and harmful to all life forms. The plant cell wall forms a physical barrier against environmental stress and changes in the cell wall structure have been observed upon Cd exposure. In the current study, changes in the cell wall composition and structure of *Medicago sativa* stems were investigated after long-term exposure to Cd. Liquid chromatography coupled to mass spectrometry (LC-MS) for quantitative protein analysis was complemented with targeted gene expression analysis and combined with analyses of the cell wall composition.

**Results:**

Several proteins determining for the cell wall structure changed in abundance. Structural changes mainly appeared in the composition of pectic polysaccharides and data indicate an increased presence of xylogalacturonan in response to Cd. Although a higher abundance and enzymatic activity of pectin methylesterase was detected, the total pectin methylation was not affected.

**Conclusions:**

An increased abundance of xylogalacturonan might hinder Cd binding in the cell wall due to the methylation of its galacturonic acid backbone. Probably, the exclusion of Cd from the cell wall and apoplast limits the entry of the heavy metal into the symplast and is an important factor during tolerance acquisition.

**Electronic supplementary material:**

The online version of this article (10.1186/s12870-019-1859-y) contains supplementary material, which is available to authorized users.

## Background

Anthropogenic influence has led to an accumulation of cadmium (Cd) in the upper soil. Originating from fertilizer application, mining activity and sewage sludge, the concentration of Cd in the topsoil ranges from < 0.01 to 14.1 ppm throughout Europe [1]. Cadmium has a very high mobility and can enter plants via their root system, from where it is distributed to all plant parts and can cause multiple toxicity symptoms. Accumulation in the above-ground parts limits the economical valorisation of plant material but on the contrary, plants can also be used to remove Cd from the soil in the phytoremediation process.

The plant cell wall is a dynamic structure, which continuously undergoes changes to adapt to the plant’s development as well as environmental conditions and serves as a physical barrier against environmental threats such as Cd. Its structural components provide mechanical support and rigidity, which is furthermore maintained by the activity of embedded cell wall proteins conferring optimal characteristics to the cell wall [2, 3]. The cell wall is mainly composed of cellulose, hemicellulose and pectins. Cellulose is the main structural component and composed of β-1,4-linked glucose, forming crystalline microfibrils. Those microfibrils are embedded in a complex, heterogeneous polysaccharide matrix. Hemicelluloses bind to cellulose and include xyloglucans, glucomannans, xylans and mixed-linkage glucans, whereby their interaction with cellulose highly contributes to cell wall strengthening [4]. Pectins are probably the most heterogeneous group of polysaccharides and include homogalacturonan (HG), rhamnogalacturonans (RG) I and II, as well as xylogalacturonans (XGA). The backbone of pectin is composed of unbranched galacturonic acid (GalA), which can be partially decorated with various sugar moieties such as xylose. As an exception, the backbone of RGI does not only contain GalA but disaccharide repeats. Galacturonic acid can be modified by methylesterification and/or acetylation, which affects the physicochemical properties of pectin. Thereby, the pectic polysaccharide homogalacturonan is highly methylated when it is build-in into to the cell wall and gets demethylated afterwards by cell wall-located pectin methylesterase (PME). However, attached sugar moieties at the galacturonic acid backbone such as xylose interfere with the accessibility of enzymes to their GalA target side [5]. Cell wall domains with a high content in XGA are characterised by a high methylation degree [6] as these patches are most likely resistant to PME activity.

**Fig. 1:**
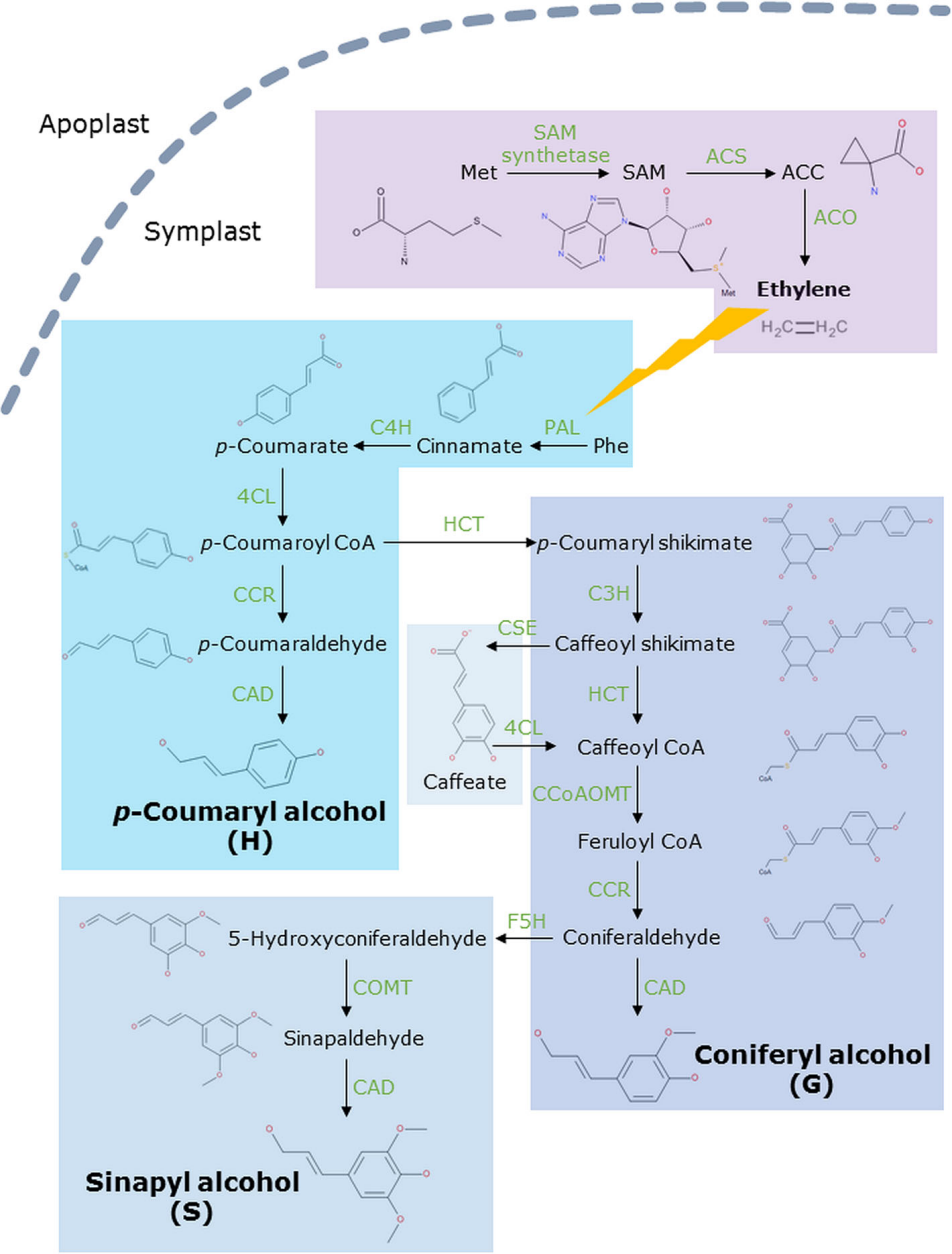
Monolignol and ethylene biosynthesis pathways. Molecules and enzymes of the pathways are indicated. Met methionine; SAM *S*-adenosylmethionine; ACC 1-aminocyclopropane-1-carboxylic acid; ACS ACC-synthase, ACO ACC-oxidase; Phe phenylalanine, PAL phenylalanine ammonia lyase; C4H cinnamate-4-hydroxylase; C3H coumarate 3-hydroxylase; HCT hydroxycinnamoyl transferase; 4CL 4-coumarate ligase; CAD cinnamyl alcohol dehydrogenase; CSE caffeoyl shikimate esterase; CCoAOMT caffeoyl-CoA 3-*O*-methyltransferase; CCR cinnamoyl CoA reductase; COMT caffeate *O*-methyltransferase; F5H ferulate 5-hydroxylase. Ethylene influences the activity of PAL (indicated as a lightning bold) and thereby affects monolignol biosynthesis. The enzyme CSE together with 4CL bypasses the second HCT reaction and appears to be important in the lignin biosynthetic pathway of *M. truncatula* [28]

During Cd exposure, alterations in the cell wall structure occur and changes in the methylation pattern of HG were observed [7, 8]. An increased PME activity and an enhanced accumulation of *PME* transcripts following Cd exposure were shown in flax [9, 10]. The de-methylesterification of HG creates binding sites for Ca, which can be displaced by Cd due to a higher affinity of the latter [11]. Thereby, the possible sequestration of Cd in the cell wall prevents its further entry into the cell and is part of the plant’s defence strategy against Cd stress [12]. Additionally, Cd induces the activity of peroxidases enhancing cell wall lignification [9, 10, 13], which is observed in different plant species [14, 15]. By inducing the accumulation of reactive oxygen species (ROS), of which hydrogen peroxide (H_2_O_2_) acts as a signalling molecule and triggers secondary reactions such as peroxidase activity, Cd contributes to cell wall lignification followed by growth inhibition [16–18]. Lignin is build up by blocks of monolignols, namely *p*-coumaryl alcohol, sinapyl alcohol as well as coniferyl alcohol and a set of different enzymes is required for their biosynthesis [19, 20] (Fig. 1). In the initial step in the synthesis of phenolic compounds such as monolignols, phenylalanine (Phe) is converted to cinnamic acid by phenylalanine ammonia-lyase (PAL), whose activity is enhanced by the gaseous plant hormone ethylene [21]. Ethylene is involved in multiple molecular, biological and physiological processes in the plant’s life cycle and has a simple biosynthetic pathway (Fig. 1). Exposure to heavy metals affects ethylene biosynthesis as well as signalling as reviewed by Keunen et al. [22]. In Cd-exposed *Arabidopsis thaliana* plants an increased concentration of the ethylene precursor 1-aminocyclopropane-1-carboxylic acid (ACC) was found and ethylene responsive genes were upregulated [23]. Furthermore, inhibition of ethylene synthesis results in a decreased PAL activity and a lower lignin content in cucumber roots [24].

*Medicago sativa* is an important forage legume worldwide and its stem tissue is often used in research to study processes taking place at the cell wall [25, 26] as it represents more than 50% of the produced biomass and is rich in cell wall material. Furthermore, the stems can be used for industrial applications such as bioethanol production, which increases the economic value of the plant. Environmental conditions such as heavy metal exposure influence the structure and composition of the cell wall impacting its valorisation potential. Thus, studies addressing the structural changes of the cell wall are of high economic and societal interest. A previous gel-based study revealed changes in the cell wall proteome of *M. sativa* stems when plants are exposed to Cd [27]. Several proteins involved in cell wall remodelling and carbohydrate metabolism were shown to be altered in their abundance, which supports the hypothesis that Cd influences the cell wall structure and underlines its function as a defence barrier against Cd stress.

This study focuses on the cell wall monosaccharide composition and lignin content in *M. sativa* stems, when plants were exposed to a realistic Cd concentration in the soil for an entire season. Based on the hypothesis that the cell wall acts as a defence barrier against Cd, the present study complements a previous gel-based study by using a gel-free approach and different hypotheses on the changed cell wall structure upon Cd exposure are addressed by targeted analysis.

## Results

### Label-free quantitative proteome analysis

A LC-MS based quantitative protein analysis was carried out on cell wall proteins, extracted in three fractions, and soluble proteins. The used targeted approach results in a cell wall protein-enrichment in which contamination with cytosolic proteins is low. However, proteins involved in photosynthesis are found throughout the three cell wall protein fractions. Cadmium interferes with photosynthetic activity and a consistent impact on the abundance of chloroplastic proteins appeared in our data. Therefore, proteins involved in photosynthesis will be included in the results and discussion.

**Fig. 2:**
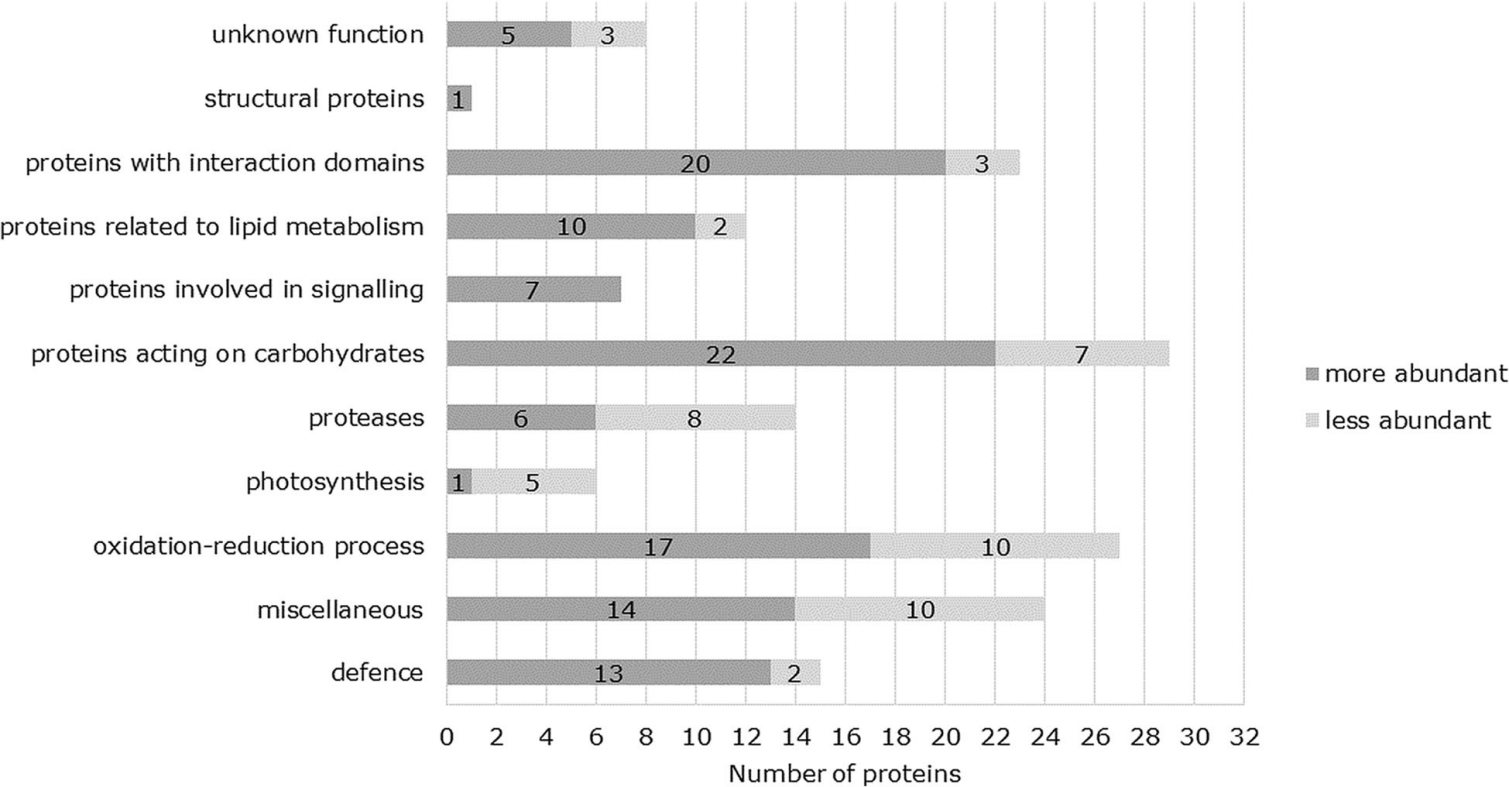
Functional classification of proteins from all three fractions (CaCl_2_, EGTA, LiCl) that changed significantly in *M. sativa* stems exposed to Cd. Quantification was done using the PROGENESIS QI software for proteomics (NonLinear Dynamics)

A total number of 166 significantly changed proteins were identified in the three cell wall fractions. Those proteins were categorized as proposed in [29] but keeping defence proteins and proteins involved in photosynthesis in separate categories (Additional file 1). Clearly more proteins were of higher abundance in response to Cd (116 proteins versus 50 proteins being of less abundance) (Fig. 2).

Looking at each cell wall protein fraction separately (Additional file 1), the highest number of proteins that changed significantly was found in the EGTA and LiCl fractions (65 and 68 respectively). While only five proteins were of less abundance due to Cd exposure in the EGTA fraction, 27 proteins in the LiCl fraction had a decreased abundance after Cd exposure. In the CaCl_2_ fraction, 33 proteins showed a significant abundance change, of which 15 were more abundant. Although the used extraction approach is targeting cell wall protein, obtained extracts are an enrichment of cell wall proteins in which the contamination with proteins of other subcellular localisations is low. Among the proteins that changed significantly, the percentage of secreted, cell wall localized proteins was high in all three fractions: CaCl_2_ fraction 66.67%, EGTA 81.46% and LiCl 77.94%.

Strikingly, a high number of proteins that have an increased abundance after Cd exposure have a designated function in plant defence. These include chitinases and chitin-binding proteins (grouped as carbohydrate binding), pathogenesis-related thaumatin family proteins, allergen Pru proteins and Kunitz type trypsin inhibitor proteins (grouped as proteins with interaction domain). Only two defence-related proteins identified in the LiCl fraction were of lower abundance (*M. truncatula* homolog of drug resistance transporter-like ABC domain protein and LRR and NB-ARC domain disease resistance protein). Different peroxidases were identified in the category oxidation-reduction process and a higher abundance in response to Cd exposure was determined in the EGTA and LiCl fraction. Contrary to this, a lower abundance of peroxidase class III (contig 34,984) was found in the LiCl fraction and CaCl_2_ fraction. Clustering of the identified peroxidases (Clustal Omega) and sequence comparison did not reveal a separation and no specific function was found for those peroxidases that are of lower abundance. Other proteins of the oxidation-reduction process which were less abundant are ferritin, multi-copper oxidase-like protein, basic blue-like protein, L-ascorbate oxidase, plastocyanin-like domain protein and early nodulin-like protein and were identified in the CaCl_2_ and LiCl fractions. Most of the proteins classified as proteins acting on carbohydrates are involved in the structure of the plant cell wall. Most prevalent, glucan endo-1,3-beta-glucosidase was identified in all three fractions and showed an increased abundance upon Cd exposure. Furthermore, alpha-galactosidase-like protein and expansin A10 in the CaCl_2_ fraction and glycoside hydrolase family 18 protein and pectinacetylesterase family protein in the EGTA fraction had an increased abundance. Other proteins of higher abundance which are known to be involved in the assembly of the cell wall are dirigent-like proteins (categorized as miscellaneous), polygalacturonase inhibiting protein 1, pectinesterase/pectinesterase inhibitor, xyloglucanase-specific endoglucanase inhibitor protein (all categorized as proteins with interaction domains), extensin-like proteins (structural protein) and FASCICLIN-like arabinogalactan-proteins (categorized as involved in signalling). Most of them were found in the EGTA and LiCl fractions. In the latter fraction, several cell wall organizing proteins were also found to be of less abundance such as beta-galactosidase-like protein and polygalacturonase non-catalytic protein, but also proteins which are of higher abundance were identified such as alpha-glucosidase, pectinacetylesterase family protein, expansins and pectinesterase/pectinesterase inhibitor. Additionally, polygalacturonase inhibitor protein in the CaCl_2_ fraction (contig 93,293) was also identified to be less abundant.

In the soluble protein fraction a total number of 28 significantly changed proteins were identified of which 25 were more abundant and three proteins had a decreased abundance (see Additional file 1 for all identifications). Of those proteins, 57.14% had no predicted target site and can be considered as cytosolic. Six proteins were predicted to be secreted and another six proteins were chloroplastic. The majority of the identified proteins are miscellaneous, small proteins involved in translation, nucleotide binding or protein folding. Furthermore, a rhicadhesin receptor, a glutamine synthase and a sieve element occlusion protein were identified. Those proteins were increased due to Cd exposure and only a 60S ribosomal L4-like protein (contig 101,331) was found to be less abundant. More abundant proteins are categorized as acting on carbohydrates (fructose-bisphosphate aldolase, glycoside hydrolase family 1 protein, glucosamine-6-phosphate isomerase/6-phosphogluconolactonase), defence (pathogenesis-related thaumatin family protein), oxidation-reduction process (nodulin-like protein, peroxidases), photosynthesis (thylakoid rhodanese-like) and proteases (eukaryotic aspartyl protease). In addition, a peroxisomal NAD-malate dehydrogenase 2 and photosystem II subunit Q-2 were of less abundance due to Cd exposure.

### Gene expression analysis

**Fig. 3:**
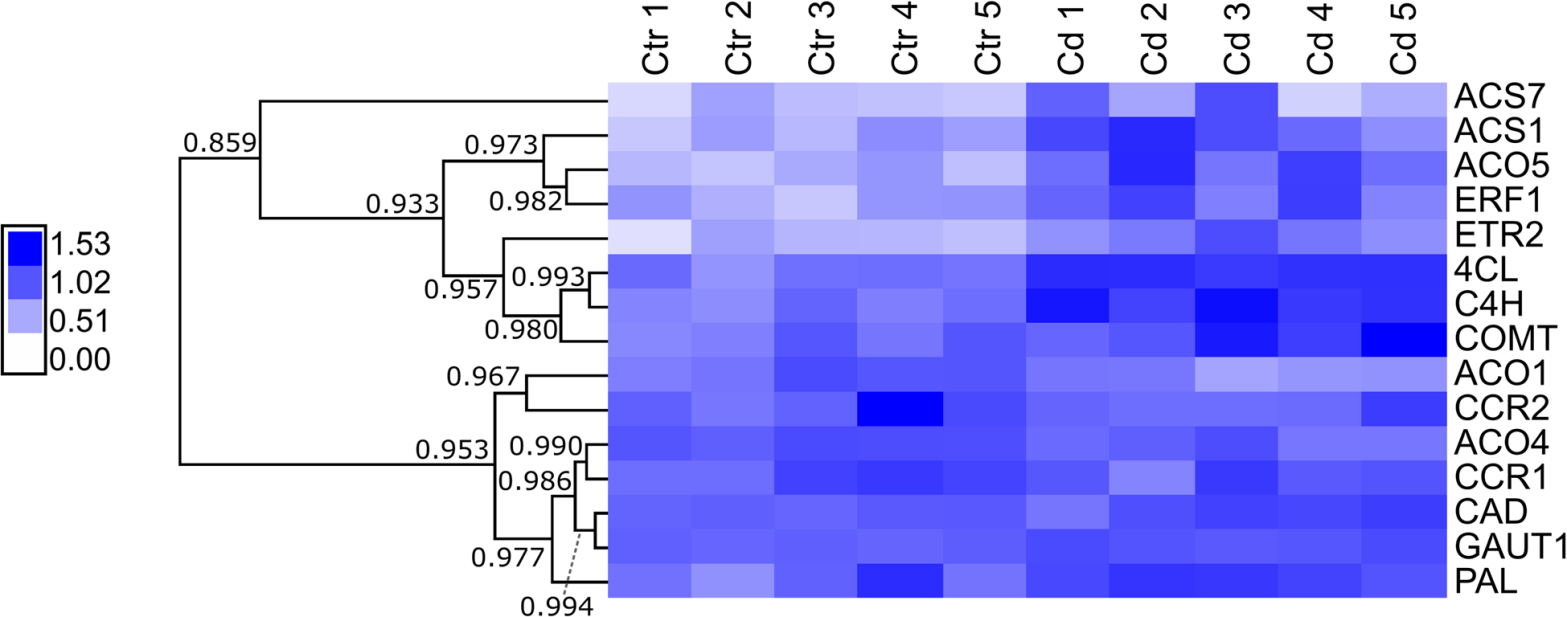
Heat map hierarchical clustering showing the expression of genes assessed by qPCR. Values represent relative normalised expression values. The colour bar indicates the expression values as an increasing intensity gradient. Numbers refer to the Pearson correlation coefficient between the lengths of two branches. ACS (1-aminocyclopropane-1-carboxylic acid synthase), ACO (1-aminocyclopropane-1-carboxylic acid oxidase), ERF (ethylene responsive factor), ETR (ethylene-resistant), 4CL (4-coumarate ligase), C4H (cinnamate-4-hydroxylase), COMT (caffeate O-methyltransferase), CCR (cinnamoyl-CoA reductase), CAD (cinnamyl alcohol dehydrogenase), PAL (phenylalanine ammonia lyase)

The expression of genes involved in monolignol, pectin and ethylene syntheses was investigated in *M. sativa* stems to reveal the impact of Cd exposure (see Fig. 1 for abbreviations of gene names). Five biological replicates were used in this experiment and relative normalised expression values were calculated as means with the standard error of the means (SEM) (Table 1). A heat map hierarchical clustering shows the expression of all genes and replicates assessed by qPCR (Fig. 3). Two patterns of gene expression were observed. In the upper branch, a higher gene expression due to Cd exposure was measured for *ACS7*, *ACS1*, *ACO5*, *ERF1* and *ETR2*. With the exception of *ACS7*, those changes of expression are significant (Table 1). Furthermore, *4CL*, *C4H* and *COMT* are found in that upper branch but the expression difference between control plant and Cd-exposed plants is low. While *4CL* and *C4H* change significantly, expression of *COMT* does not (Table 1). Genes, which cluster in the lower branch, have a lower expression in Cd-exposed plants but in general the expression values show a high variation between replicates in the same condition (Fig. 3). Only the expression change of *ACO1* and *ACO4* is significant (Table 1). In a second group of the lower branch cluster *CAD*, *PAL* and *GAUT1*. The latter is a 1,4-galacturonyltransferase that synthesises HG [30]. While *GAUT1* is significantly higher expressed in Cd-exposed plants, great variations between replicates can be seen for *CAD* and *PAL*. Nevertheless, mean expression values of the three genes indicate a higher expression in response to Cd exposure (Table 1).

**Table 1:**
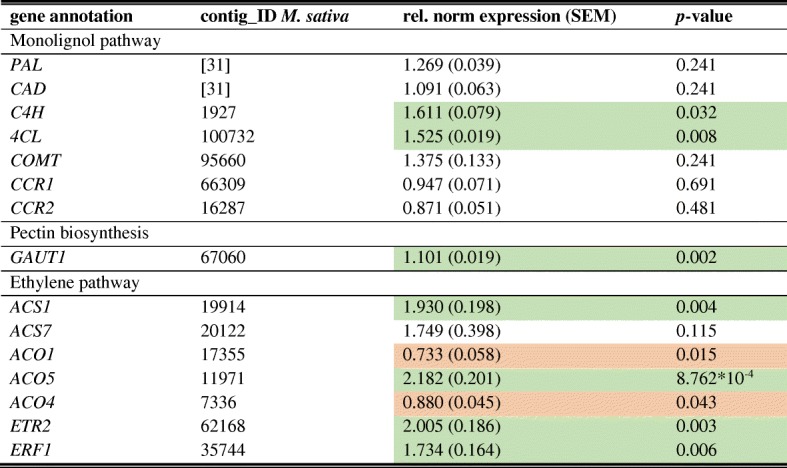
Relative normalised gene expression in *M. sativa* stems

Normalised expression values are expressed relative to the control set at 1.00. Values are given as an average of 5 replicates with the standard error of the mean (SEM). A *t*-test was done to determine the significance (*p* ≤ 0.05). Green: significantly upregulated by Cd; red: significantly downregulated by Cd

### Monosaccharide composition in the stem cell wall after long-term exposure to cd

**Fig. 4:**
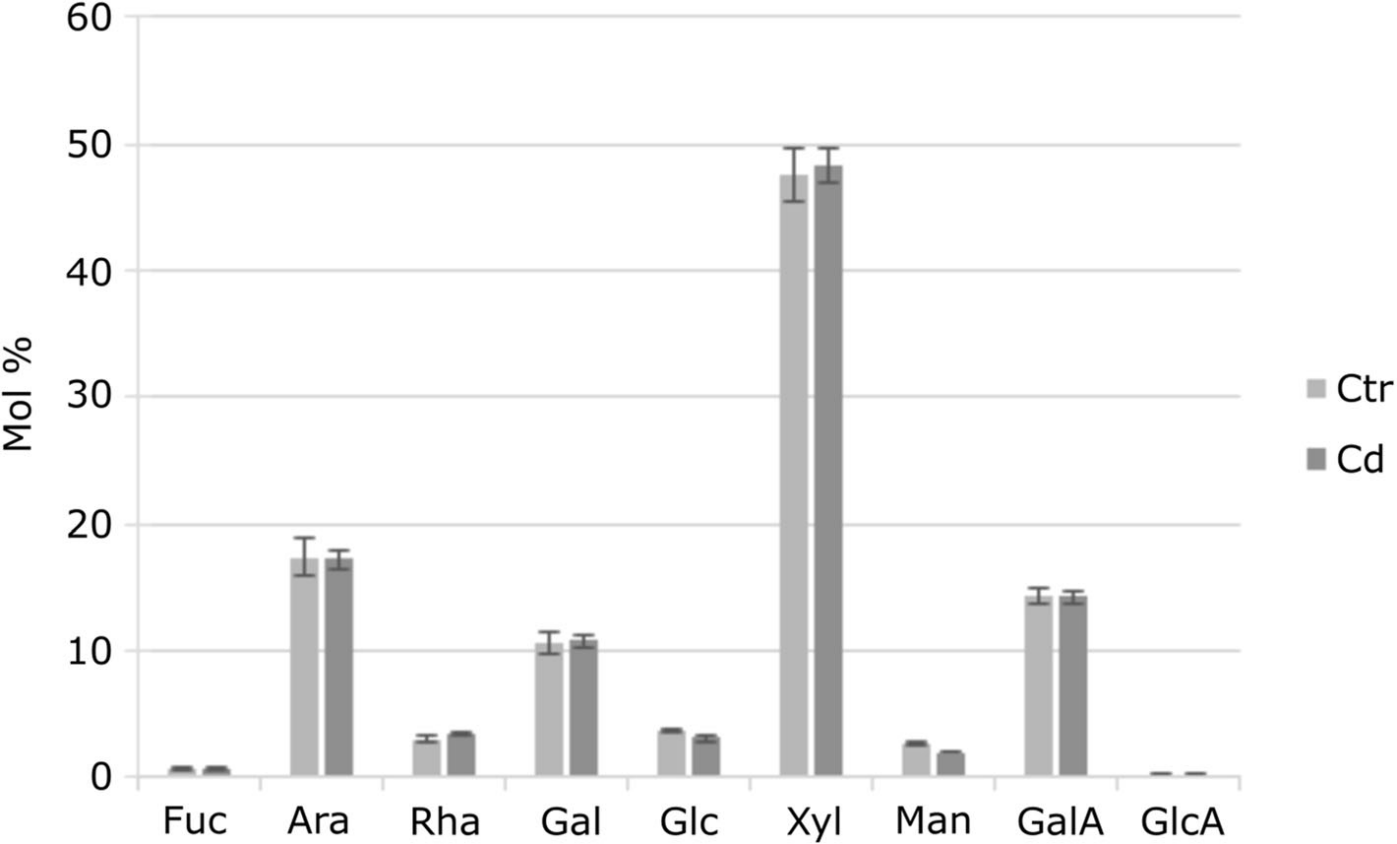
Monosaccharide composition of total cell wall material of stems from Cd-exposed and control *M. sativa* plants. Given values are the average of five biological replicates. Error bars indicate the SEM

The total sugar composition of the cell wall from *M. sativa* stems was determined after TFA hydrolysis (Fig. 4). This analysis excludes crystalline cellulose as it is TFA resistant. The most prominent monosaccharide was by far xylose (Xyl), which represents almost 50% in Cd-exposed (48.34%) and control plants (47.46%). The next high abundant monosaccharides were, in decreasing order, arabinose (Ara), galacturonic acid (GalA) and galactose (Gal). The lowest abundance was determined for fucose (Fuc), rhamnose (Rha), glucose (Glc), mannose (Man), and glucuronic acid (GlcA). No difference in the global monosaccharide composition between Cd-exposed and control plants was observed.

**Fig. 5:**
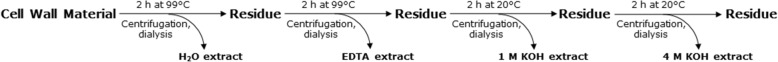
Workflow for the sequential extraction of monosaccharides from cell wall material

The prepared total cell walls from Cd-exposed and control *M. sativa* stems were fractionated by sequential extraction (Fig. 5). At the end of the sequential extraction, a final cell wall pellet (CWP) remained, which was also hydrolysed and the monosaccharide composition was analysed (Table 2).

**Table 2:** Monosaccharide composition of the different sequential extracts of *M. sativa* stems

Average mol percentage (mol %)										
Fraction		Fuc	Ara	Rha	Gal	Glc	Xyl	Man	GalA	GlcA
H_2_O	Ctr	0.45 ± 2.74^−2^	36.63 ± 1.46	1.96 ± 1.16^−1^*	22.49 ± 4.22^− 1^	12.30 ± 7.37^− 1^	4.07 ± 3.01^− 1^*	7.75 ± 7.06^− 1^	13.70 ± 1.2	0.67 ± 9.04^− 2^
	Cd	0.50 ± 1.87^− 2^	34.56 ± 8.14^− 1^	2.53 ± 1.69^− 1^*	23.55 ± 4.86^− 1^	10.54 ± 3.06^− 1^	5.21 ± 3.21^− 1^*	7.99 ± 3.95^− 1^	14.61 ± 7.07^− 1^	0.51 ± 6.16^− 2^
EDTA	Ctr	0.81 ± 2.13^− 2^	52.81 ± 1.48*	3.65 ± 1.34^− 1^*	15.94 ± 2.66^− 1^	1.96 ± 7.97^− 2^*	4.77 ± 2.91^− 1^*	4.16 ± 1.38^− 1^	15.37 ± 1.18*	0.52 ± 6.4^− 2^
	Cd	0.77 ± 2.47^− 2^	44.67 ± 6.82^− 1^*	4.26 ± 2.2^− 1^*	15.77 ± 3.23^− 1^	1.68 ± 4.82^− 2^*	6.61 ± 1.02^–1*^	4.27 ± 1.69^− 1^	21.30 ± 6.99^− 1^*	0.68 ± 1.71^− 1^
1 M KOH	Ctr	0.23 ± 2.29^− 2^	4.21 ± 4.25^− 1^	1.18 ± 1.18^− 1^	2.32 ± 2.64^− 1^	2.37 ± 2.95^− 1^	73.11 ± 9.95^− 1^	1.10 ± 8.91^− 2^	15.29 ± 2.5^− 1^	0.20 ± 9.69^− 3^
	Cd	0.25 ± 3.2^−2^	4.28 ± 5.36^− 1^	1.49 ± 2.05^− 1^	2.63 ± 3.54^− 1^	2.05 ± 2.61^− 1^	71.36 ± 1.67	1.69 ± 4.05^− 1^	16.04 ± 8.22^− 1^	0.21 ± 3.74^− 2^
4 M KOH	Ctr	1.76 ± 7.38^− 1^	6.23 ± 2.63	0.44 ± 1.53–^1^*	5.60 ± 5.12^− 1^*	10.74 ± 6.21	53.50 ± 2.56	11.67 ± 3.92	9.41 ± 1.93	0.65 ± 5.6^− 1^
	Cd	1.65 ± 3.41^−2^	6.21 ± 1.25^− 1^	0.70 ± 7.72^− 2^*	6.30 ± 5.68^− 2^*	15.01 ± 7.45^− 1^	49.81 ± 2.11	11.78 ± 2.09	7.72 ± 5.24^−1^	0.82 ± 2.15^− 1^
CWP	Ctr	0.54 ± 1.58^−2^	11.93 ± 3.18^−1^*	1.65 ± 5.74^− 2^*	13.63 ± 2.97^− 1^	20.50 ± 3.78^− 1^	36.52 ± 7.1^− 1^	3.62 ± 1.06^− 1^*	11.17 ± 2.17^− 1^	0.43 ± 2.8^− 2^
	Cd	0.60 ± 4.26^− 2^	17.00 ± 1.76*	2.14 ± 1.18^− 1^*	14.54 ± 1.44	20.72 ± 2.08	30.58 ± 4.8	2.84 ± 1.86^− 1^*	11.22 ± 5.11^− 1^	0.37 ± 5.98^− 2^

SEM standard error of the mean, Ctr control, Fuc fucose, Ara arabinose, Rha rhamnose, Gal galactose, Glc glucose, Xyl xylose, Man mannose, GalA galacturonic acid, GlcA glucuronic acid; CWP cell wall pellet

Values are given as an average from five biological replicates ± SEM. Significant changes were determined with a *t*-test (*p*-value ≤0.05) and are indicated with an asterisk

All analysed monosaccharides were present in the H_2_O fraction, mostly Ara, followed by Gal, GalA, Glc, Man, Xyl, Rha and traces of GlcA and Fuc. Slight significant differences between the cell walls of Cd-exposed and control plants appeared only for Rha and Xyl. Ara and Gal were the most abundant neutral monosaccharides in the EDTA fraction. The co-extraction of Rha suggests the presents of RGI in this fraction with side chains of arabinan and/or arabinogalactan, whereby Ara was significantly lower abundant in the Cd-exposed stem cell wall, while the abundance of Rha increased. GalA represents the pectin backbone and its content significantly increased about 5.9% in the cell wall of Cd-exposed stems. Furthermore, the co-extraction of Xyl with GalA indicates the presence of xylogalacturonan in the cell wall of *M. sativa* stems and a significantly increased abundance of Xyl was reported due to Cd exposure. In the KOH fractions, Xyl was found to be the most abundant monosaccharide. The 1 M KOH fraction contained mainly Xyl (more than 70% in both conditions), followed by GalA. However, both were not affected by Cd-exposure. Besides the great portion of Xyl, the 4 M KOH fraction contained all other analysed monosaccharides, to the least proportion Rha and GlcA. Some differences in the presence of monosaccharides (Gal, Glc, Xyl, GalA) appeared between exposed and non-exposed plants, of which only Gal significantly changed. Furthermore, a slight variation was detected for Rha, which is significant. The final pellet, which remained at the end of the sequential extraction was also hydrolysed with TFA. This fraction still contained high amounts of Xyl, Glc, Gal, Ara and GalA. Thereby, a significantly higher proportion of Ara and Rha was present in the remaining pellet of Cd-exposed plants. On the other hand, Man significantly decreased in Cd-exposed plants.

### PME activity and pectin methylation

**Fig. 6:**
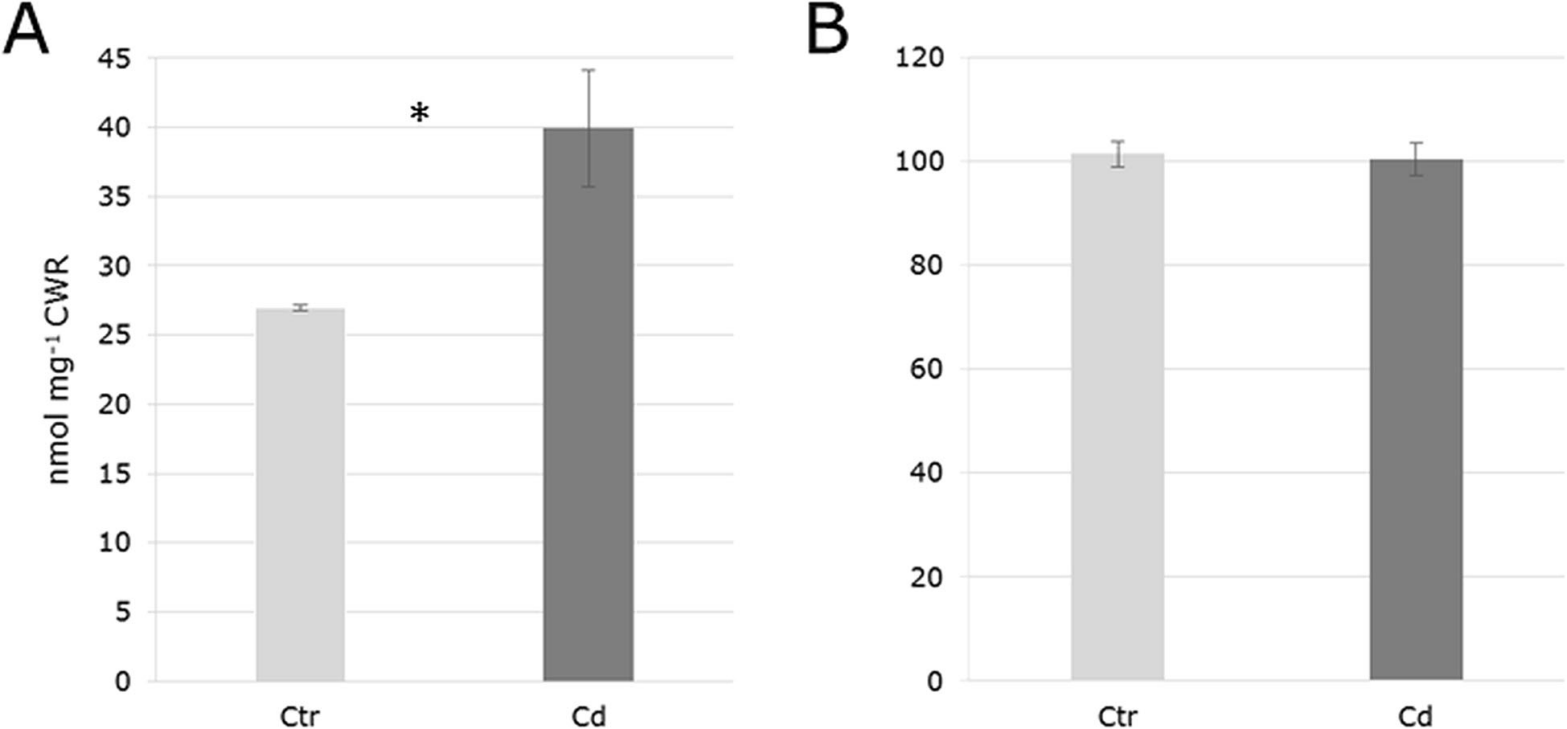
Methanol released (nmol mg^− 1^ CWR) by pectin methylesterase (PME) activity (**a**) and saponification with 1 M KOH (**b**) from the cell wall of *M. sativa* stems in response to long-term Cd exposure. Values represent mean values from five replicates, each measured in technical replicates. SEM is represented as error bars. Significance (*p*-value 0.05) is indicated by an asterisk

The PME activity and pectin methylation was assessed in five biological replicates of which each was measured in two technical replicates. PME activity was significantly increased in Cd-exposed samples as was monitored by means of the methanol produced (nmol mg^− 1^ CWR) (Fig.5 a) and reflects the accumulation of the proteins in response to Cd exposure (Additional file 1). However, the amount of released methanol from the cell wall after saponification was the same for control and Cd-exposed samples and Cd exposure did not appear to alter the pectin methylation degree despite the determined increased PME activity (Fig. 6b).

### Analysis of lignification in stem after long-term exposure to cd

The total lignin content was determined spectrophotometrically and compared between stems of Cd-exposed plants and plants grown on unpolluted soil after long-term exposure. No difference in the lignin concentration appeared. The composition of lignin in the CWR was assessed after nitrobenzene oxidation and covered the main lignin degradation products *p*-hydroxybenzaldehyde (H), vanillin (V) and syringaldehyde (S). The monolignol composition did not change between conditions. In fact, a high variability in the monomeric lignin composition was observed between replicates (Table 3).

**Table 3:** Lignin content and monomer composition of *M. sativa* stems

	Lignin (% CWR)	H (μmol g^− 1^ CWR)	V (μmol g^− 1^ CWR)	S (μmol g^− 1^ CWR)
Ctr	4.68	10.95 (3.01)	527.50 (275.10)	336.18 (145.77)
Cd	4.70	14.57 (2.61)	545.60 (172.55)	338.48 (103.22)

Given values are the average of five replicates with the standard error of the mean (SEM) in parentheses. Ctr control, CWR cell wall residues, H *p*-hydroxybenzaldehyde, V vanillin, S syringaldehyde

### Quantification of ethylene precursor molecules in response to long-term cd exposure

**Fig. 7:**
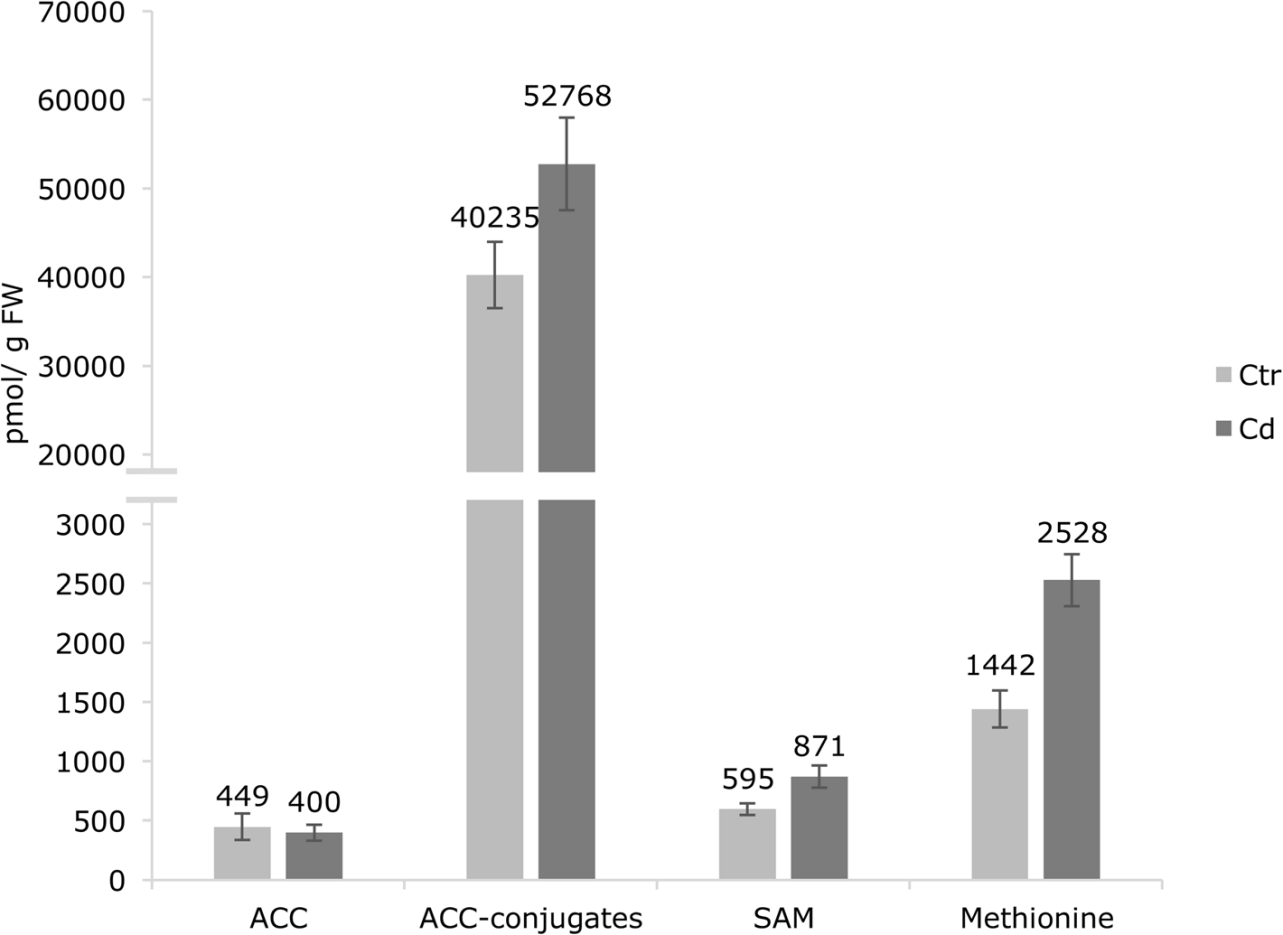
Content of precursor molecules of ethylene biosynthesis (pmol g^− 1^ FW) in stems of *M. sativa* after long-term Cd exposure. Data are given as means from five biological replicates. Error bars indicate the SEM. Significance was determined with a t-test (*p* ≤ 0.05) and is indicated with an asterisk

To evaluate the effects of Cd on ethylene synthesis, concentrations of ACC and its conjugates were determined in Cd-exposed and unexposed plants (Fig. 7). As the immediate precursor, the presence of ACC determines a rate-limiting step in ethylene biosynthesis and eventually influences the hormone concentration [32]. Long-term Cd exposure did not affect the ACC content significantly but an enormous increase of conjugated ACC was observed (about 31.15%), which is however not significant. Furthermore, *S*-adenosylmethionine (SAM) and methionine content were significantly increased about 46.39 and 75.31% respectively upon Cd exposure (Fig. 7).

## Discussion

For the current study, *M. sativa* plants were grown on Cd-contaminated soil (10 mg kg^− 1^ soil DW) for an entire season (May till September). As it is agricultural practice, plants were cut and the secondary stem was sampled after a re-growing stage. Although strong growth inhibition occurred in a juvenile plant stage, no difference in biomass was measured when sampling them [27]. The present study investigates how Cd exposure changes the composition and structure of the cell wall. Previous data which were based on a gel-based approach [27] are complemented with a gel-free approach using the same samples and different hypotheses on the changed cell wall structure upon Cd exposure are addressed by targeted analyses.

To target cell wall proteins, extraction buffers with increasing ionic strength were used successively to extract step by step also tightly bound proteins. Several proteins, which were found of higher abundance in the CaCl_2_ and EGTA fraction, appeared in the LiCl fraction to be of lower abundance (Additional file 1). The LiCl buffer has the highest ionic strength and therefore extracts the more tightly bound cell wall proteins. Structural alterations in the cell wall might influence cell wall - protein interactions, thereby causing shifts of a protein in between the different fractions. This type of shifts in affinity due to changes in the cell wall can influence the interpretation during quantification. This observation could be a first indication for structural changes that appear in the cell wall in response to Cd. Changes in the composition and properties of cell wall polysaccharides can alter the function of the cell wall during stress response, thereby protecting the plant from severe Cd-induced damages.

However, as was observed in a gel-based study on *M. sativa* stems [27], long-term Cd exposure led to a higher abundance of defence-related proteins such as different chitinases, pathogenesis-related thaumatin family proteins as well was glucan endo-1,3-beta-glucosidase (Additional file 1), which reflects the general stress response of the plant and appears to be rather unspecific.

### The effect of long-term cd exposure on the cell wall composition in *M. sativa* stems

The plant cell wall is composed of cellulose as the main load-bearing polysaccharide, hemicelluloses and pectins. Its composition and remodelling are involved in the responses to abiotic stress [33, 34]. Thereby, cell wall proteins, which are embedded in the polysaccharide network and represent about 10% of the cell wall, can act on the cell wall structure and customise the cell wall properties during development and environmental stresses such as Cd exposure. The here presented cell wall protein data (Additional file 1) revealed significant changes in abundance of several proteins upon long-term Cd exposure with a function in cell wall structure and these findings are in agreement with a previous study [27].

In the total extraction of monosaccharides from isolated cell wall material of *M. sativa* stems, no significant differences between control and Cd-exposed plants appeared (Fig. 4). Under both growing conditions the main monosaccharide is Xyl. Already in 1987 Xyl was found to be the most prominent in mature *M. sativa* stems, enhancing their indigestibility by cross-linking with lignin [35].

The cell wall polysaccharides were furthermore sequentially extracted with H_2_O, EDTA and KOH (1 M followed by an extraction with 4 M) to obtain a more comprehensive picture of the cell wall composition. Overall, results are comparable with those previously obtained from *M sativa* stems of the same developmental stage [36].

Pectic polysaccharides were extracted with the chelating agent EDTA. Galacturonic acid, the building block for HG, was the main pectic residue, while glucuronic acid was present only in trace amounts (Table 2). The GalA of HG can be decorated with residues of xylose forming domains of xylogalacturonan (XGA), a pectic polysaccharide which was demonstrated to be present in the cell wall of various tissues of *Arabidopsis* [37]. The co-extraction of Xyl with GalA in the EDTA fraction indicates the presence of XGA in the cell wall of *M. sativa* (Table 2)*.* Xylogalacturonan is a commonly found component in the cell wall of legumes [38] and cell walls of soy bean are rather composed of XGA and RG than of HG [6]. It was demonstrated that XGA is present in regions of cell detachment [39]. Here, the amount of extracted GalA and Xyl significantly increased in response to Cd exposure (Table 2), suggesting a higher abundance of XGA in the cell wall due to the applied stress. The HG backbone of XGA can be partially methylated. The de-methylation of HG by PME is a key process during the modulation of the pectin network and its activity facilitates the clustering of pectin to a gel-like matrix [40]. The decoration of HG with Xyl inhibits gel formation as it hinders the accessibility of pectin-modifying enzymes to their target sites [5, 41] and stabilises the high methylation degree of pectin [42]. Nonetheless, Cd-exposure increased the abundance and enzyme activity of PME in *M. sativa* stems (Fig. 6a, Additional file 1), which would decrease the methylation degree of the cell wall and promote Cd binding within the pectin network. However, data did not reveal changes in the methanol released from the cell wall fraction upon saponification between control and Cd-exposed plants (Fig. 6b). The methylesterification degree of pectin in the cell wall can vary within a tissue leading to local differences of the mechanical cell wall properties even on a small spatial scale [43]. The XGA incorporation into the cell wall of *M. sativa* stems will certainly affect the gel properties of the wall matrix. There are probably regions with less XGA, which are accessible for the PME and its higher abundance and enzyme activity in response to Cd (Fig. 6a, Additional file 1) could compensate for the XGA-rich areas in order to maintain a particular overall de-methylation degree of the cell wall. These domains in the cell wall network could serve for Cd demobilization, while those domains with a higher portion of XGA are protected from the PME activity.

Studies suggest that heavy metal tolerance of plants is determined by the degree of pectin methylation and make the link to heavy metal sensitivity or resistance. Heavy metal tolerant populations were found to have a higher degree of pectin methylation [44]. Consequently, this limits the ability of the cell wall to bind heavy metals and maintains a low apoplastic heavy metal concentration, resulting into a decreased symplastic uptake. Both processes are an important factor for the tolerance of the plant [45]. Thus, a XGA-rich cell wall can help to exclude Cd from the apoplast, which is combined with a local Cd deposition in the cell wall of *M. sativa* stems. In that way, the here observed structural alterations in the cell wall contribute to the previously reported tolerance of *M. sativa* to long-term Cd exposure [27].

Most hemicelluloses were extracted with KOH of different concentrations. The most prevalent monosaccharide was xylose and the determination of Fuc in both KOH fractions indicate to xyloglucan. The co-extraction of GalA in the KOH fractions (Table 2) may result from a cross-linkage between pectin and xyloglucan [46]. Xyloglucan is widely spread throughout the cell wall of plants [47]. Its linkage to pectin highly contributes to the cell wall structure and assembly and support the integration of xyloglucan into the cell wall [48].

However, different glycosyl hydrolase family proteins were of higher abundance (Additional file 1). Those are known to be involved in cell wall deconstruction, loosening and growth [49, 50]. Apparently, the growth during stressful conditions is a conflict between stiffening by cross-linking and loosening of the cell wall. While ROS and peroxidases support stiffening by cross-linkage of hemicellulose with lignin, expansins and glycosyl hydrolases support loosening [34].

### Long-term cd exposure affects ethylene biosynthesis but not lignification and monolignol composition

Ethylene is a gaseous hormone which is involved in the response to multiple biotic and abiotic stresses [22, 51, 52] and its levels increased after metal exposure in different plants [23, 53]. In the current study, long-term Cd exposure led to increasing content of methionine and SAM. Next, SAM is converted to ACC by the activity of ACC synthase (ACS). The transcript levels of both investigated gene isoforms *ACS1* and *ACS7* were upregulated in stems of Cd-exposed *M. sativa* plants, suggesting an enhanced production of ACC, which is the rate-limiting step in ethylene biosynthesis [32]. While ACC content slightly decreased upon Cd exposure, the abundance of its conjugated forms increased, although this change is insignificant (*p* = 0.086). The three known conjugates of ACC are γ-glutamyl-ACC (GACC), jasmonyl-ACC (JA-ACC) and malonyl-ACC (MACC) and conjugation might be a biochemical regulation of available ACC which affects ethylene levels [54]. The synthesis of MACC positively correlates with ethylene production, suggesting that a self-regulated feedback control of ethylene synthesis is active which would stimulate the storage of ACC [55]. Possibly MACC can be converted back to ACC thereby stimulating the ethylene production [56].

In a final step, ACC is oxidised to ethylene by ACC oxidase (ACO) and in the current study the decrease of ACC in Cd-exposed plants was accompanied by an upregulation of *ACO5* transcript levels and of the ethylene responsive genes *ETR2* and *ERF1*. Therefore, free ACC gets converted to ethylene, enhancing ethylene signalling in response to long-term Cd exposure.

Besides other mechanism [22], ethylene positively affects the activity of PAL [21, 24], thereby augmenting the lignin content [57]. The PAL enzyme catalyses the conversion of Phe to cinnamate, making the entry into monolignol biosynthesis. Those monolignols are the building blocks for lignin biosynthesis. The impact of Cd on cell wall lignification and plant growth is well established [14, 58, 59] and changes in the lignin composition in the context of abiotic stress were observed [60]. Nevertheless, after long-term Cd-exposure of *M. sativa* plants, lignin content of the cell wall and transcript level of *PAL* did not significantly change. Furthermore, the monolignol composition was not affected after long-term Cd exposure either (Table 3). Yet, a significantly changed accumulation of *C4H* and *4CL* transcripts were detected; both encoding enzymes of the monolignol biosynthetic pathway (Table 1). Cadmium-induced stimulation of PAL activity was shown to be dose and time dependent in roots of *Matricaria chamomilla*, which also applies for the content of soluble phenolics and their accumulation [61]. In the current study, the applied Cd concentration was kept low to mimic realistic soil concentrations and could have been too low to significantly affect lignin biosynthesis and content, although the ethylene biosynthesis was induced accompanied by a variation of *PAL* transcript levels.

Lignification is driven by peroxidase activity. Quantitative LC-MS revealed an increased abundance of different peroxidase isoforms due to Cd-exposure as observed before [27]. Peroxidases and their activity increase upon Cd exposure [62] and therefore lead to increased lignification [59]. For lignin formation, peroxidases require H_2_O_2_ as a substrate molecule. Furthermore, Cd induces oxidative stress in plant cells and enhances the generation of H_2_O_2_ [63]. However, no difference in the lignification between stems of control and Cd-exposed plants was detected in the current study. Probably those peroxidases are more involved in H_2_O_2_ scavenging during long-term mild Cd-exposure instead of altering the lignin content [34, 64]. During LC-MS analysis, a decreased abundance of some peroxidase isoforms was observed. After clustering all identified isoforms in a phylogenetic tree, no separation of the less abundant isoforms from the more abundant isoforms appeared (data not shown). Thus, the observed decrease of the identified peptides might be due to degradation fragments as Cd also induces proteolysis which is underlined by an increased abundance of proteases monitored in this study (Additional file 1).

## Conclusion

Long-term Cd exposure led to an adaptation of *M. sativa* to the applied stress. Phenotypically, no difference was observed between Cd-exposed and control plants in their mature growing stage, which is reflected by similar biomasses. During long-term Cd exposure the composition of pectic polysaccharides in the cell wall of *M. sativa* stems changes. Against the initial hypothesis that the cell wall undergoes structural changes supporting the immobilization of Cd, data indicate an increased abundance of XGA upon Cd exposure. Contrary to HG, XGA is resistant to PME activity and therefore stabilises the high methylation degree of pectin. Thus, the creation of binding sites for Cd in the pectin network does not take place. Probably, the exclusion of Cd from the cell wall and apoplast limits the entry of the heavy metal into the symplast, which is an important factor during tolerance acquisition. As another important aspect for the cell wall restructuring, no increase in lignification was reported, although peroxidases are highly abundant in Cd-exposed plants. Most likely peroxidases are accumulating as a response to Cd-induced oxidative stress in order to maintain the redox balance in *M. sativa*. However, long-term Cd exposure stimulated ethylene signalling in *M. sativa* stems. The plant hormone positively influences the activity of PAL and the phenolpropanoid pathway. PAL is not only induced by ethylene but further depends on substrate availability [65] and the Phe content is a key-factor in the process of cell wall lignification and cell wall stiffening. Understanding how this is influenced by long-term Cd exposure in *M. sativa* stems would be valuable to model the plant response to the applied stress.

## Methods

### Plant material and sampling

*Medicago sativa* L. seeds, cultivar Giulia, were inoculated with *Sinorhizobium meliloti* and sown on Cd-contaminated and control soil. The soil was prepared as one batch composed of 2/3 potting soil mixed with 1/3 sand (w/w). Half of the prepared soil was contaminated with Cd applied as CdSO_4_ to a final concentration of 10 mg Cd per kg soil dry weight (DW). Plants were sown in May 2015 in 12 times 12 pots for each condition. The plants were kept in the greenhouse until the flowering stage was reached and subsequently cut similar to the agricultural practice. For the re-growing period, the plants were kept outside till the pre-flowering stage was reached followed by one more week in the greenhouse before the final sampling (10th of September 2015). During the entire experiment no fertilizer was applied nor was the day-cycle or temperature controlled. The first and the last two internodes were removed from the stems and only the middle parts were sampled to obtain a more homogeneous sample. Stems were sampled in five biological replicates for each condition, with a pool of stem material from 24 pots corresponding to one biological replicate. Samples were ground to a fine powder in liquid nitrogen and kept at − 80 °C till further use.

### Label-free quantitative proteome study

#### Protein extraction and preparation

Cell wall and soluble protein extraction of five replicates from *M. sativa* stems were done as described before [66, 67].

Digestion of proteins was performed using an Amicon Ultra-4 10 K Centrifugal filter device (Millipore) [68]. Cell wall and soluble proteins, 20 μg of each sample, were reduced with 10 mM DTT in 100 mM ammonium bicarbonate (AmBic) for 20 min and subsequently washed with 100 mM AmBic (30 min, 4700 g, 4 °C). Reduced samples remained on top of the filter and were alkylated with 50 mM iodoacetamide dissolved in 100 mM AmBic for 30 mins in the dark. After two washing steps, samples were digested with 40 μL trypsin Gold (Promega), 5 ng ml^− 1^ trypsin in 20 mM AmBic, at 40 °C overnight. Afterwards, 100 μL H_2_O was added on the filter, devices were centrifuged (40 min, 4700 g, 4 °C) and peptides collected from the bottom of the tube. The peptides were dried under vacuum and solubilized in 40 μL of 5% acetonitrile (ACN) and 0.01% trifluoroacetic acid (TFA).

#### LC-MS/MS peptide separation and analysis

Peptides were analysed with a NanoLC-2D System (Eksigent) coupled to a TripleTOF 5600+ MS (Sciex) as was previously detailed in Behr et al. 2018 [69]. The CID spectra were analysed with Mascot-Daemon (version 2.4.2, Matrix Science) by searching against the alfalfa EST database downloaded from the Samuel Roberts Noble website (675,750 sequences; 304,231,702 residues, released on 3rd of November 2015) [70] with the following parameter settings: 2 missed cleavages, mass accuracy precursor: 20 ppm, mass accuracy fragments: ± 0.5 Da, fixed modifications: carbamidomethyl (C), dynamic modifications: Oxidation (M and P), Acetyl (protein N-term), Didehydro (F) and tryptophan to kynurenine. To ensure proteins identifications at least two assigned peptides needed to pass the MASCOT-calculated score of ≥25 and the peptides should have been identified in at least 80% of the replicates. Mascot data were imported in PROGENESIS QI software for proteomics (NonLinear Dynamics) for quantitative analysis. Quantitative results were statistically evaluated by means of a one-way ANOVA *p*-value (*p* ≤ 0.05) as well as a fold-change of 1.5 to reveal proteins with a significantly different abundance. In the quantitative analysis only unique peptides were considered. Proteins, for which a significant change was observed, were manually validated to avoid false positive identifications. The subcellular location was determined by the TargetP online tool [71] using standard parameters. Thereby, proteins were considered as cell-wall targeted when a secretion signal peptide was predicted or the subcellular localization was found based on literature. Identified cell wall proteins were categorised into functional classes following Duruflé et al. 2017 [29].

### Real time quantitative PCR (qPCR)

The RNAqueouse™ Kit (Life Technologies) was used for RNA extraction from five biological replicates according to the manufacturer’s instructions. The RNA was purified with 3 M sodium acetate and 100% isopropanol, subsequently washed with 70% ethanol before resuspention in RNase-free water. A NanoDrop® ND-1000 spectrophotometer (Thermo Fischer Scientific) was used to determine the concentration and quality (A_260/280_ and A_260/230_ ratio between 1.9 and 2.5). One μg of the extracted RNA was DNase treated (TURBO DNA-free™ Kit, Life Technologies) and reverse transcribed (PrimeScript™ RT Reagent Kit; Perfect Real Time, TAKARA Bio Inc.). The obtained cDNA was diluted 10-fold in 1/10 Tris-EDTA buffer (Sigma-Aldrich) and stored at − 20 °C till further use.

The Alfalfa Gene Index and Expression Atlas Database [70] was used to design specific primer pairs for genes of interest by using open source tools (www.bioinformatics.nl/cgi-bin/primer3plus/primer3plus.cgi, https://eu.idtdna.com/calc/analyzer). Primer pairs for PAL and CAD were taken from literature [31]. The primer efficiency was evaluated prior to gene expression analysis (Additional file 2: Table S1). All qPCR reactions were performed in a 96 well plate with the 7500 Fast Real Time PCR System (Life Technologies) as described elsewhere [27]. All details according to Minimum Information for publication of Quantitative real-time PCR Experiments [72] are shown in Additional file 2: Table S2. Gene expression was calculated according to the 2^-∆Cq^ method relative to the sample with the highest expression. Obtained data were normalised using the average 2^-∆Cq^ values of the three most stable reference genes which were selected by the GrayNorm algorithm [73] out of ten tested [31].

All values were expressed relative to the control samples. The significance was assessed with a *t*-test with *p*-value ≤0.05. Normalised relative expression data were clustered (uncentered Pearson correlation, complete linkage) [74] and displayed as a heat map [75].

### Isolation of cell wall residues

A sufficient amount of powdered deep frozen plant material from five biological replicates was mixed with 40 mL 80% methanol, sonicated for 10 min and shaken for 4 h at room temperature. The homogenates were subsequently centrifuged (3700 g) and the pellet washed five times with 80% ethanol by a vortexing/centrifugation cycle. The isolated cell wall residues (CWRs) were dried (45 °C, 24 h) and served as material to analyse lignin, the cell wall composition, the determination of the PME activity and pectin methylation.

### Sequential extraction of monosaccharides from the stem cell wall

Isolated CWRs (90 mg) were incubated in 1.5 mL of 0.1 M acetate buffer (pH 5.0) for 20 min at 80 °C followed by digestion with 10 μL α-amylase plus 10 μL amyloglucosidase shaken overnight at 37 °C to remove the starch from the samples. The reaction was stopped by the addition of 6 mL 100% cold ethanol. Samples were kept at − 20 °C for 3 h, subsequently washed in 100% ethanol three times and dried at room temperature. From the isolated cell wall material, 15 mg were used for the sequential extraction, whereby the supernatant was kept after each extraction step and the remaining pellet was used for the next extraction step (Fig. 5).

The first extraction step was done three times with 1.5 mL water at 99 °C, 1000 rpm for 2 h and contained water-soluble polysaccharides. The second extraction was done three times with 0.1% EDTA (pH 7.5) at 99 °C, 1000 rpm for 2 h to solubilise pectins. The third and fourth extractions were done with 1 M potassium hydroxide (KOH) and 4 M KOH respectively with addition of 10 mM sodium borohydride (NaBH_4_). Both extraction steps were incubated for 2 h at 20 °C, 1000 rpm and extracted mainly hemicelluloses. Prior to dialysis, the pH of 1 M and 4 M KOH fractions was neutralized with 75 μL and 300 μL of acetic acid respectively.

Float-A-Lyzer® G2 devices (Spectrum Laboratories) with a molecular weight cut-off of 0.5–1.0 kD were used to dialyze the extracts. The devices were washed with 10% ethanol and H_2_O prior to dialysis according to the manufacturer’s instructions. All dialysis steps were done under constant stirring. The 4 M KOH fraction was first dialyzed against a neutralized 1 M KOH solution (2 h) and subsequently transferred in a neutralized 0.25 M KOH solution together with the 1 M KOH fraction (2 h). Finally, all four fractions were dialyzed overnight against H_2_O and freeze-dried (Alpha 1–4 LD plus, Christ). The isolated CWR, the four freeze-dried fractions and the final remaining pellet from the sequential extraction were hydrolysed at 99 °C in 500 μL 2 M TFA for 2.5 h. Samples were cooled down on ice, centrifuged (10,000 g, 3 min) and the supernatant was used for analysis in two-times and 100-times dilutions.

Quantitative results were obtained by Ionic Chromatography coupled to Pulsed Amperometric Detection (ICS 5000+, Thermo-Dionex). The voltage of the gold electrode was kept at 0.1 V for 0.4 s, reduced to − 2 V within 0.02 s, increased to 0.6 V within 0.01 s, reduced to − 0.1 V for 0.07 s. The AgCl electrode was used as a reference. Two chromatographic methods were applied to separate all monosaccharides. The eluents were prepared from ultra-pure water and sodium hydroxide (NaOH). For the first method, 5 μL were injected on a CarboPac PA20 column (3 × 150 mm + guard column 3 × 30 mm, Thermo-Dionex) at 30 °C (0.5 mL min^− 1^). The gradient started with 12 mM NaOH for 10 min, increased to 300 mM within 2 min and was kept at 300 mM for 5 min. For the second method, 1 μL was injected on a CarboPac SA10-4 μm column (2 × 250 mm + guard column 2 × 50 mm, Thermo-Dionex) at 45 °C (0.38 mL min^− 1^). The gradient started with 1 mM NaOH for 5 min, increased to 198 mM within 3 min and was kept at these conditions for 2 min.

### PME activity assay

The PME activity assay was done as described before [76]. Any methanol that is released by the PME activity is converted into formaldehyde by alcohol oxidase. In a second reaction, formaldehyde forms a complex with purpald, resulting in a purple coloration. A standard curve was generated by using methanol at different concentrations. Ten mg of prepared CWR were suspended in 200 μL 1 M NaCl and shaken for 1 h at 4 °C. Samples were subsequently centrifuged (13,200 g, 10 min, 4 °C) and the supernatant recovered for the PME activity assay. One reaction contained 100 μL pectin in PBS (0.64 mg mL^− 1^), 10 μL alcohol oxidase (0.01 U μL^− 1^) and 50 μL PME sample. The reaction was incubated at 30 °C for 10 min, subsequently 200 μL purpald (5 mg mL^− 1^ in 0.5 N NaOH) was added and the mixture was incubated for 30 min at 30 °C. Finally, 550 μL H_2_O was added and the absorption measured at 550 nm. For both conditions, five biological replicates were analysed and measured in two technical replicates.

### Methylation of pectin

Hydrolyses of methylated pectin and determination of resulting methanol was done as descripted before [77] with slight modifications. Briefly, 300 μL of 1 M KOH were added to five mg of isolated CWR and incubated for 30 min at room temperature. The pectin hydrolysates were neutralized (7.0 pH) with 1 M phosphoric acid and the volumes adjusted to 1 mL. For the determination of methanol present in the extract, the same protocol as for the PME activity assay was followed.

### Extraction and characterization of lignin in stem cell wall

Five mg of CWR were digested with 2.6 mL of 25% acetyl bromide in glacial acetic acid for 2 h at 50 °C using a HACH LT200 system. Samples were cooled on ice and transferred into 10 mL of 2 M NaOH plus 12 mL glacial acetic acid. The reaction tube was rinsed with glacial acetic acid and 1.75 mL of 0.5 M hydroxylammonium chloride was added. Each volume was adjusted to 30 mL with glacial acetic acid, centrifuged (3000 g, 15 min) and lignin content was measured spectrophotometrically (280 nm, extinction coefficient ε = 22.9 g^− 1^ L cm^− 1^).

Lignin was characterized following the nitrobenzene oxidation method previously described [69, 78]. The products after nitrobenzene oxidation of ten mg of CWR were derivatized with Bis(trimethylsilyl)trifluoroacetamide and analysed by gas chromatography coupled to mass spectrometry (GC-MS). A HP-5MS column (30 m × 0.25 mm, 0.25 μm, Agilent) was used on a 7890B-5977A GC-MS system (Agilent). Salicylic acid-D4 was used as internal standard.

### Determination of compounds of the ethylene biosynthetic pathway

Extraction was done in 500 μL ice-cold 80% methanol from 50 mg finely ground plant material. For quantification, D4-ACC (250 pmol, Olchemim Ltd.) and D3-Methionine (1000 pmol, Sigmar-Aldrich) was added. Half a milligram of OASIS HLB 0.3 μm solid phase bulk packing material (WATERS) was added to bind pigments. The packing material and cell debris were removed with centrifugation (14,000 g, 4 °C, 10 min). The ACC metabolites and precursors were analysed with ES^+^ UPLC-MS/MS (ACQUITY TQD, WATERS) using a Waters Column ACQUITY UPLCr BEH Amide 1.7 μm Column. The eluents were 0.1% formic acid in H_2_O (A) and 0.1% formic acid in ACN (B). The gradient was as following: gradient 0–2 min: 15.0% A, 85.0% B; 2–5.8 min: linear gradient to 35.0% (A), 65.0% B; 5.8–6.4 min: linear gradient to 80.0% A, 20.0% B; isocratic at 80.0% A, 20.0% B until 7 min. The flow rate was 0.4 mL min^− 1^. The partial loop injection mode was used with an injection volume of 6 μL. The specific transitions selected for multiple reaction monitoring (dwell time 0.034 s. for each transition) were: 106.10 > 60.20 (cone: 14.0, collision energy 10.0) and 106.10 > 88.00 (cone 14.0, collision 8.0) for D4-ACC; 150.00 > 104.00 (cone 15.0, collision 15.0) for methionine; 153.00 > 107.00 (cone 14.0, collision 10.0) for D3-Methionine; 189.00 > 130.00 (cone 16.0, collision 12.0) for malonyl-ACC; 232.00 > 148.00 (cone 16.0, collision 12.0) for glutamyl-ACC; 295.00 > 148.00 (cone 16.0, collision 12.0) for jasmonyl-ACC and 399.40 > 250.00 (cone 16.0, collision 15.0) for SAM. The quantity of ACC was analysed by CI^−^ GC-MS/MS after derivatization using pentafluorobenzyl bromide following [79]. Data are expressed in picomoles per gram fresh weight (pmol g FW^− 1^).

## Additional files

Additional file 1: Quantitative LC-MS/MS data and protein assignments. Overview of the proteins that changed significantly in the different cell wall protein fractions. (XLSX 95 kb)

Additional file 2: **Table S1.** Sequence information on forward and reverse primers used to determine gene expression levels via quantitative real-time PCR. **Table S2.** Quantitative real-time PCR parameters according to the MIQE guidelines. (DOCX 32 kb)

## Data Availability

The mass spectrometry proteomics data have been deposited to the ProteomeXchange Consortium via the PRIDE [80] partner repository with the dataset identifier PXD009670 and 10.6019/PXD009670

## Abbreviations

4CL: 4-coumarate ligase
ACC 1: Aminocyclopropane-1-carboxylic acid
ACN: Acetonitrile
ACO: ACC-oxidase
ACS: ACC synthase
AmBic: Ammonium bicarbonate
Ara: Arabinose
C3H: Coumarate 3-hydroxylase
C4H: Cinnamate-4-hydroxylase
Ca: Calcium
CAD: Cinnamyl alcohol dehydrogenase
CCoAOMT: Caffeoyl-CoA 3-O-methyltransferase
CCR: Cinnamoyl CoA reductase
Cd: Cadmium
COMT: Caffeate O-methyltransferase
CSE: Caffeoyl shikimate esterase
Ctr: Control
CWP: Cell wall pellet
CWR: Cell wall residues
DW: Dry weight
F5H: Ferulate 5-hydroxylase
Fuc: Fucose
Gal: Galactose
GalA: Galacturonic acid
GC-MS: Gas chromatography coupled to mass spectrometry
Glc: Glucose
GlcA: Glucuronic acid
HCT: Hydroxycinnamoyl transferase
HG: Homogalacturonan
KOH: Potassium hydroxide
LC: Liquid chromatography.
Man: Mannose
Met: Methionine
MS: Mass spectrometry
NaOH: Sodium hydroxide
PAL: Phenylalanine ammonia-lyase
Phe: Phenylalanine
PME: Pectin methylesterase
ppm: parts per million
RG: Rhamnogalacturonan
Rha: Rhamnose
ROS: Reactive oxygen species
SAM: S-adenosylmethionine
SEM: Standard error of the means
TFA: Trifluoroacetic acid
XGA: Xylogalacturonan
Xyl: Xylose
