# Supplementary material for: Does long-term cadmium exposure influence the composition of pectic polysaccharides in the cell wall of Medicago sativa stems?
Source: BMC Plant Biol. 2019 Jun 21;19:271. doi: 10.1186/s12870-019-1859-y (PMC6588869; doi:10.1186/s12870-019-1859-y)
Supplement: Supplementary file 2 — Table S1. Sequence information on forward and reverse primers used to determine gene expression levels via quantitative real-time PCR. Table S2. Quantitative real-time PCR parameters according to the MIQE guidelines. (DOCX 32 kb) [file 12870_2019_1859_MOESM2_ESM.docx]

**Additional file 2:**

**Table S1.** Forward (Fw) and reverse (Rev) primers used to determine gene expression levels via quantitative real-time PCR. The Alfalfa Gene Index and Expression Atlas Database was used to obtain the coding sequences for the genes of interest which are given as contig numbers.

| Annotation | Contig | Sequence (5’-3’) | Amplicon (bp) | Efficiency (%) |
| --- | --- | --- | --- | --- |
| phenylalanine ammonia lyase (PAL) | Guerriero et al., 2014 [27] | Fw: ATGAGGTGAAGCGTATGGTG | 141 | 94 |
|  |  | Rev: CATCCCTAGCAGATTCAGACAG |  |  |
| cinnamyl alcohol dehydrogenase (CAD) | Guerriero et al., 2014 [27] | Fw: TTCAAAGACTTTGGCCGAGG | 88 | 99 |
|  |  | Rev: TGCCACCATTGTTGGGTTAAG |  |  |
| cinnamate-4-hydroxylase (C4H) | 1927 | Fw: CTTGAAAGCACCAAGAGCAC | 82 | 94 |
|  |  | Rev: CACCCTTCTTTTGAGCATCC |  |  |
| 4-coumarate ligase (4CL) | 100732 | Fw: TGAGGTGCCAGTAGCATTTG | 92 | 87 |
|  |  | Rev: ACACAACCTGTTTGGAGACG |  |  |
| caffeate *O*-methyltransferase (COMT) | 95660 | Fw: CCTCTTCGCCATGCAATTAG | 112 | 86 |
|  |  | Rev: AATTTGAGCACCAGGTCCAG |  |  |
| cinnamoyl-CoA reductase 1 (CCR1) | 66309 | Fw: CTGGGATTGTTTGTCTCATCG | 123 | 98 |
|  |  | Rev: GTCGGCACTTGAAAAAGAGG |  |  |
| cinnamoyl-CoA reductase 2 (CCR2) | 16287 | Fw: AGACGAATGTGATGCTCGTG | 101 | 94 |
|  |  | Rev: AAGGTGCCTGTTCCATCAAC |  |  |
| 1,4-galacturonyltransferase (GAUT1) | 67060 | Fw: AGAGCTTTCACCGATTCGAC  Rev: GTTCATTCCGTATGCCCAAC | 98 | 104 |
| ACC-synthtase 1 | 19914 | Fw: CTCCCAAATTCTCAACACCTC | 144 | 148 |
|  |  | Rev: GACAAAAGCTGGTGACTCTT |  |  |
| ACC-synthase 7 | 20122 | Fw: TCTCTCTCTTTCTCCTCCTTCG | 72 | 93 |
|  |  | Rev: CAACACAAGGGTGTTCTTGTTC |  |  |
| ACC-oxidase 1 | 17355 | Fw: ACATTGAGGGGAAGCTGAT | 132 | 157 |
|  |  | Rev: CAACATCACAGGTGTTCTGC |  |  |
| ACC-oxidase 5 | 11971 | Fw: ATTCAGACGATCCCACGAAG | 79 | 111 |
|  |  | Rev: TCCCTCCAATTATGCACCTC |  |  |
| ACC-oxidase 4 | 7336 | Fw: ACTGGGGATTCTTTGAGCTG | 72 | 104 |
|  |  | Rev: TGGTTAACCTCTCCACAGTGTC |  |  |
| ETR 2 | 62168 | Fw: TTGGACCTGTTGGATCTTCC | 139 | 100 |
|  |  | Rev: GCACTTGCAGTCAAGGCAAC |  |  |
| ERF 1 | 35744 | Fw: AAACGACCCAGAAGAGATGC | 131 | 104 |
|  |  | Rev: CGCCTCGATAAGATTTGTGG |  |  |
| Reference genes used for normalisation of gene expression | | | | |
| Cyclophilin | Guerriero et al., 2014 [27] | Fw: CAAACTTTCCTGACGAGTCACC | 74 | 104 |
|  |  | Rev: ACGGTCAGCAATTGCCATTG |  |  |
| GAPD | Guerriero et al., 2014 [27] | Fw: CCCTCTCCCTGTACAAAACTC | 90 | 99 |
|  |  | Rev: ACACGTAACACCAACCTTCC |  |  |
| PAB4 | Guerriero et al., 2014 [27] | Fw: GCAAGTTTACGTGGGACCCTTC | 70 | 100 |
|  |  | Rev: GTTGAATTTTGCCCTGTCACCTG |  |  |

**Table S2:** Quantitative real-time PCR parameters according to the Minimum Information for publication of Quantitative real-time PCR Experiments (MIQE) guidelines derived from Bustin *et al*. (2009) [28]. All procedures were performed according to the manufacturer’s protocols.

| **Sample/Template** | |
| --- | --- |
| Source | Stems of *Medicago sativa* plants cultivated in soil |
| Method of preservation | Liquid nitrogen |
| Storage time | 12 month at -80 °C |
| Handling | Frozen |
| Extraction method | Phenol-free Total RNA isolation: RNAqueous^TM^ Kit (Life Technologies) |
| RNA: DNA-free | TURBO DNA-free^TM^ Kit (Life Technologies)  Design of intron-spanning primers whenever possible |
| Concentration | NanoDrop®: ND-1000 Spectrophotometer (Thermo Fischer Scientific) |
| **Assay optimisation and validation** | |
| Accession number | Additional file 2 Table S1 |
| Amplicon details | Amplicon size: Additional file 2 Table S1 |
| Primer sequences | Additional file 2 Table S1 |
| *In silico* | Primer design with the Primer3Plus online tool (www.bioinformatics.nl/cgi-bin/primer3plus/primer3plus.cgi) and primer analysis with OligoAnalyzer 3.1 (https://eu.idtdna.com/calc/analyzer) |
| Empirical | A primer concentration of 100 nM was used  Annealing temperature: 60°C |
| Priming conditions | Combination of oligodT-primers and random hexamers |
| PCR efficiency | Dilution series (slope, y-intercept and r²; Additional file 1 Table S1) |
| Linear dynamic range | Samples are situated within the range of the efficiency curve |
| **Reverse transcription - PCR** | |
| Protocols | As stated in the Materials and methods section |
| Reagents | As stated in the Materials and methods section |
| No template control (NTC) | Cq and dissociation curve verification |
| **Data analysis** | |
| Specialist software | 7500 Fast Real-Time PCR System (Life Technologies)  Software v2.0.1 |
| Statistical justification | Five biological replicates  Relative expression of each gene was calculated as an average after elimination of outliers  Log transformation of the data  Significance of changes in gene expression was determined by a *t*-test at a significance level of 0.05 using Microsoft Excel |
| Normalisation | Three reference genes were selected as described in the Materials and methods section |
